# Systematic comparison of somatic variant calling performance among different sequencing depth and mutation frequency

**DOI:** 10.1038/s41598-020-60559-5

**Published:** 2020-02-26

**Authors:** Zixi Chen, Yuchen Yuan, Xiaoshi Chen, Jiayun Chen, Shudai Lin, Xingsong Li, Hongli Du

**Affiliations:** School of Biology and Biological Engineering, South China University of Technology, Guangzhou, 510006 China

**Keywords:** Data processing, Data processing, Mutation, Mutation, DNA sequencing

## Abstract

In the past decade, treatments for tumors have made remarkable progress, such as the successful clinical application of targeted therapies. Nowadays, targeted therapies are based primarily on the detection of mutations, and next-generation sequencing (NGS) plays an important role in relevant clinical research. The mutation frequency is a major problem in tumor mutation detection and increasing sequencing depth is a widely used method to improve mutation calling performance. Therefore, it is necessary to evaluate the effect of different sequencing depth and mutation frequency as well as mutation calling tools. In this study, Strelka2 and Mutect2 tools were used in detecting the performance of 30 combinations of sequencing depth and mutation frequency. Results showed that the precision rate kept greater than 95% in most of the samples. Generally, for higher mutation frequency (≥20%), sequencing depth ≥200X is sufficient for calling 95% mutations; for lower mutation frequency (≤10%), we recommend improving experimental method rather than increasing sequencing depth. Besides, according to our results, although Strelka2 and Mutect2 performed similarly, the former performed slightly better than the latter one at higher mutation frequency (≥20%), while Mutect2 performed better when the mutation frequency was lower than 10%. Besides, Strelka2 was 17 to 22 times faster than Mutect2 on average. Our research will provide a useful and comprehensive guideline for clinical genomic researches on somatic mutation identification through systematic performance comparison among different sequencing depths and mutation frequency.

## Introduction

Cancer is one of the major diseases that threaten human health. It is estimated that there are approximately 18.1 million new cancer cases and 9.6 million cancer deaths in 2018^1^. Due to cancer heterogeneity, the same treatment approach could result in huge efficacy differences for different individuals with the same type of cancer^2,3^. Therefore, no therapy can be universally applied to all cancers so far and more precise therapies should be developed. In recent years, cancer treatments have made great progress, especially targeted therapy. Currently, most targeted cancer therapies are based on detecting genes mutation. For example, some tyrosine kinase inhibitors, such as afatinib and erlotinib, are applied to target the mutation of epidermal growth factor receptor (EGFR) in non-small-cell lung cancer^4,5^. The B-Raf Proto-Oncogene, Serine/Threonine Kinase (BRAF) inhibitors, such as Sorafenib, are developed based on the trial of melanoma with the V600E mutation in BRAF^6^. Moreover, olaparib, a poly (ADP-ribose) polymerase (PARP) inhibitor, is used to treat advanced ovarian cancer with *BRCA* gene mutation^7^.

Therefore, mutation research is one of the vital steps to reveal the mechanisms of cancers and could help develop more targeted drugs. Whole-exome sequencing (WES) is an effective approach to detect genome mutations. It is reported that WES can detect 95% coding regions and >98% mutations by targeted capture chips and next-generation sequencing^8,9^. Because of its relatively low-cost, it is suitable for large cohort research and has been successfully applied to several cohort researches^10–12^.

Single-cell sequencing researches have proved that several subclones could coexist in one patient, the percentage of each subclone would be different and each subclone may have a different genetic background^13,14^. Furthermore, the pathogenic subclones may coexist with different percentages, which might result in different mutation frequency and lead to more difficulties in detecting them. The result of detecting somatic mutations can be influenced by many factors, such as sequencing depth, the proportion of pathological mutated subclone and mutation calling software.

A large number of tools are able to call somatic mutations, such as Mutect2, Varscan, Vardict, Strelka2, DeepVariant etc^15–18^. The Mutect2 tool in GATK is developed by the Broad Institute and is one of the most widely used mutation-calling tools. Strelka2 software is developed in recent years and claimed to be time-efficient, which is a very important aspect of clinical usage. There are several studies in recent literature on the performance of these mutation-calling tools^19–21^, in these studies, the overall performance of both GATK-Mutect2 and Strelka2 was stable and relatively accurate. Therefore, we choose Mutect2 and Strelka2 for somatic mutation-calling pipeline in the present study.

Up to now, which sequencing depth can provide sufficient information to detect low-frequency mutations remains to be investigated. To systematically evaluate the performance of sequencing depth and mutation frequency combinations of Strelka2 and Mutect2 tools, we conducted Illumina high-depth sequencing on two standard DNA samples (NA12878 and YH-1), the sequencing data were mapped to reference genome and duplicated reads were removed, then the data were downsampled and mixed to simulate different sequencing depths and different mutation frequency, the mixed samples were used to call somatic mutation by Strelka2 and Mutect2, respectively. Finally, the mutation-calling performance was assessed. The result of our study can provide a useful reference and guidance to obtain reliable somatic mutation using WES sequencing in clinical researches and targeted cancer therapy.

## Results

### A summary of datasets and analysis

**Figure 1:**
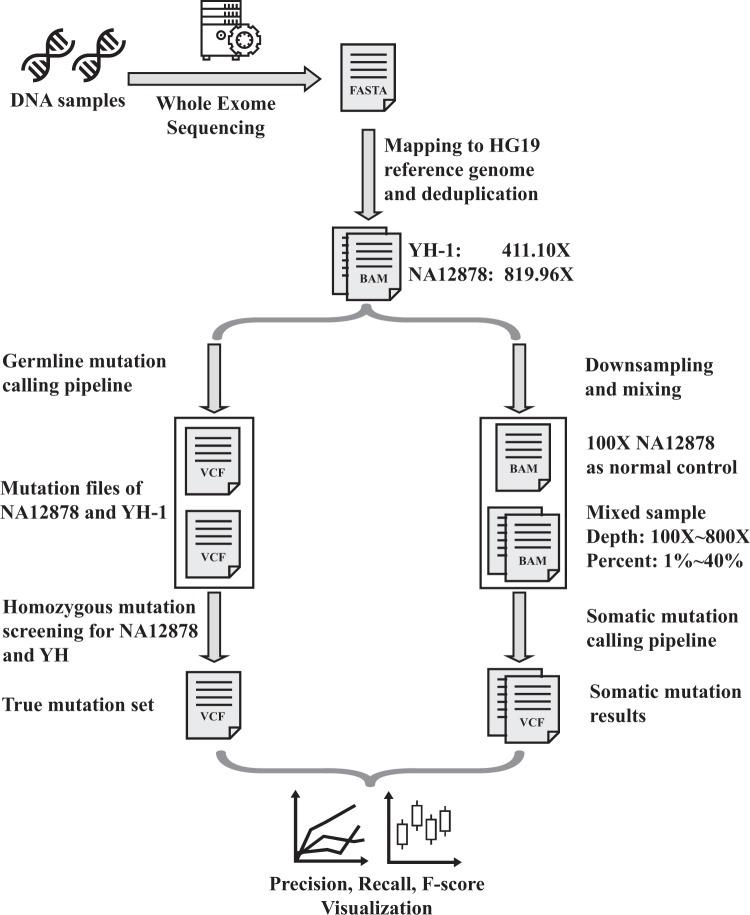
The work flowchart of the experiment design. Two DNA samples were first sent to sequencing, the sequencing reads mapped to hg19 reference genome. Then the germline mutation calling pipeline were conducted to obtain true mutation set; the high depth data were downsampled and mixed to simulate tumor samples and then conduct somatic mutation calling pipeline. The results of somatic mutation calling were then compared with true mutation set and visualized for further dissusion.

**Figure 2:**
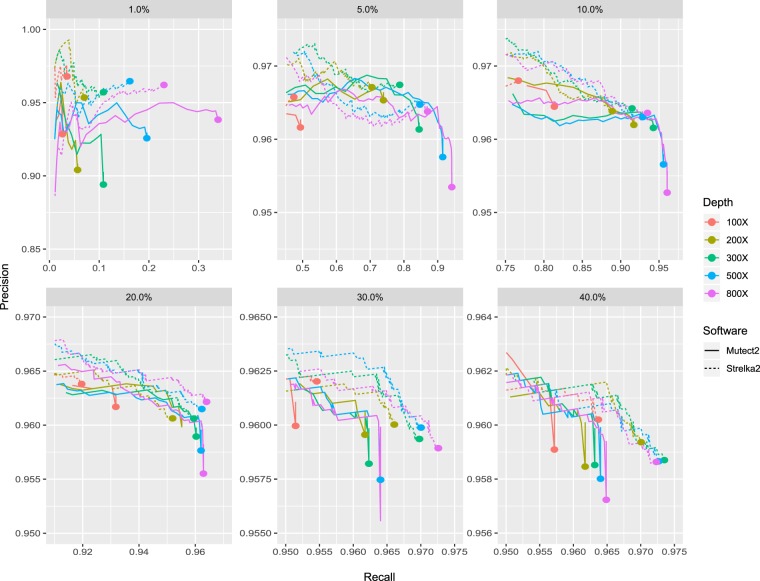
P-R curves of Strelka2 and Mutect2 for different mutation frequency and sequencing depth. The P-R curves of replicate group 1. The colors in the figure represent different sequencing depths, the dotted lines represent Strelka2 and the solid lines represent Mutect2.

The workflow of our research was presented in Fig. 1. WES-sequencing of two standard DNA samples was conducted, the detailed information of sequencing data is presented in Table 1. After obtaining the raw sequencing data, quality control was conducted, reads were mapped to the hg19 reference genome, after removing duplicated reads using Picard, the average depth of NA12878 and YH-1 was 819.96X and 411.10X, respectively. Then the NA12878 bam file was down-sampled to 100X as a normal control for the following somatic calling pipeline, and the YH-1 bam file was set as a “tumor” sample and mixed with NA12878. Different mutation frequency was simulated by controlling different YH-1 percentages in the sample mixing step. Only sites with completely different homozygous genotypes between YH-1 and NA12878 were selected into true mutations set, thus the percentage of YH-1 can be taken as the somatic mutation frequency for the mixed sample. The depths of mixed bam files were grouped into 100X, 200X, 300X, 500X and 800X, and for each depth, 1%, 5%, 10%, 20%, 30% and 40% of YH-1 was mixed with NA12878, separately. To reduce the influence of random effect in down-sampling, three replicates were generated for each depth and percentage. All 90 bam files above were used to call somatic variants by Strelka2 and Mutect2 tools, respectively. Figure 2 shows the precision-recall curves of the replicate group 1, and Supplementary Figs. S1 and S2 present curves of replicate groups 2 and 3, respectively. The recall rate, precision and F-score of three replicates and the average value of three replicates are listed in Supplementary Tables S1 and S2. Three replicate groups showed well concordance, and the differences among these replicate groups for almost all combinations were less than 2%, 3.5% and 0.022 for recall rate, precision and F-score, respectively.

**Table 1:** Detailed information of sequencing data.

Sample	Total reads(M)	Clean Reads(M)	Total base(G)	Q20 base(G)	Q30 base(G)	Q20%	Q30%	GC content	Duplication rate	Average Depth (removed duplicates)
NA12878	635.17	614.43	95.28	91.12	86.89	95.64	91.20	0.50	0.19	819.96
YH-1	298.25	292.09	44.74	42.76	41.63	95.57	93.05	0.49	0.16	411.1

### Somatic variant calling performance comparison between different sequencing depth

**Figure 3:**
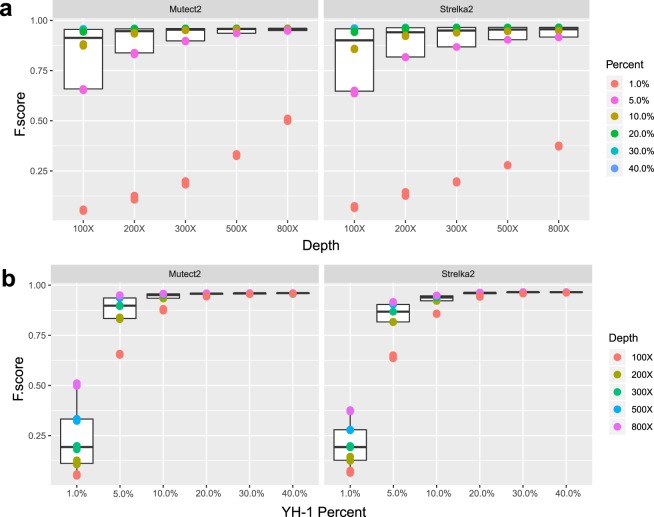
F-score box-scatter plot. The box-scatter plot of F-score, the colors represent different mutation frequency (**a**) and sequencing depths (**b**).

In order to evaluate the somatic mutation calling performance at different sequencing depth, we compared precision rate, recall rate and F-score, and drew the precision-recall curves for each mixed sample (Fig. 2, Supplementary Figs. S1 and S2). The P-R curves manifested that a higher sequencing depth could improve the recall rate, which was increased by 0.6~44% when the depth increased from 100X to 200~800X. We also observed a decrease in precision when sequencing depth was greater than 200X, and the decreased scale of precision was less than 0.7%. Furthermore, we compared F-score between different sequencing depth, the box-scatter plot presented the F-scores of all mixed samples between sequencing depths (Fig. 3a). Additionally, in concordance with P-R curves, higher sequencing depth can improve the performance of somatic mutation calling, increasing sequencing depth to 200~800X can improve the F-score by 0.02~0.45 compared with those of 100X across all mutation frequency and software (see Supplementary Table S2). In general, among all sequencing depths in our study, the result of 800X showed the best, with 23~97% recall rate, more than 93% precision, and relative highest F-score (0.374~0.96) across all mutation frequency and tools (Fig. 3b, Supplementary Figs. S1 and S2).

### Somatic variant calling performance comparison between different mutation frequency

The result of the P-R curves revealed that mutation frequency can largely influence the performance benefit of increasing sequencing depth (Fig. 2, Supplementary Figs. S1 and S2). Obviously, a low mutation frequency (1%) led to poor performance in somatic mutation calling and low recall rate (2.7~34.5%) across all depths and software (see Supplementary Table S1). For a higher proportion, the recall rate reached 48~96% and 92~97% for 5~10% and 20~40% mutation frequency, respectively. In the meantime, the precision rate was 68.9~100% across all depths and software. Figure 3b shows the F-score distribution among different mutation frequency. The F-scores were 0.05~0.51 when the mutation frequency was 1%, and they were 0.63~0.95 and 0.94~0.96 when the mutation frequency were 5~10% and 20~40%, respectively (see Supplementary Table S2).

### Somatic variant calling performance and concordance between Strelka2 and Mutect2

Considering that the choice of different tools would cause a significant effect on mutation-calling, the performance and concordance between Strelka2 and Mutect2 were further compared. In general, both Strelka2 and Mutect2 performed well when analyzing higher mutation frequency (≥20%) data, because over 90% variants were identified when precision kept greater than 95% and the F-scores ranged between 0.94 and 0.965 under these depths. The precision rate, the recall rate and the F-score of Strelka2 were slightly greater than Mutect2 but the difference between them was less than 1%. For mutation frequency of 5~10%, compared with Mutect2, the precision of Strelka2 was higher (96.2~96.5% vs 95.5~95.9%), and the recall of Strelka2 was lower (48~93% vs 50~96%), which led to the F-score of Strelka2 was lower than Mutect2 (0.64~0.94 vs 0.65~0.95). For the lowest mutation frequency (1%) data, the F-score of Strelka2 (0.06~0.19) was slightly higher than Mutect2 (0.05~0.19) when sequencing depth was 100X~300X, but the F-score of Mutet2 (0.32~0.50) surpassed Strelka2 (0.27~0.37) when sequencing depth increased to 500X and 800X (see Supplementary Tables S1 and S2).

The overall concordance of Strelka2 and Mutect2 in different sequencing depths and mutation frequency is presented in Supplementary Table 2. Generally, the concordance between Strelka2 and Mutet2 was higher than 90% except when the mutation frequency was low (1% and 5%). Concordance of the two software was 20~41% when the mutation frequency was 1%, and it was 75~89% when the mutation frequency was 5% (see Supplementary Table 3).

### Somatic variant calling efficiency of Strelka2 and Mutect2

**Figure 4:**
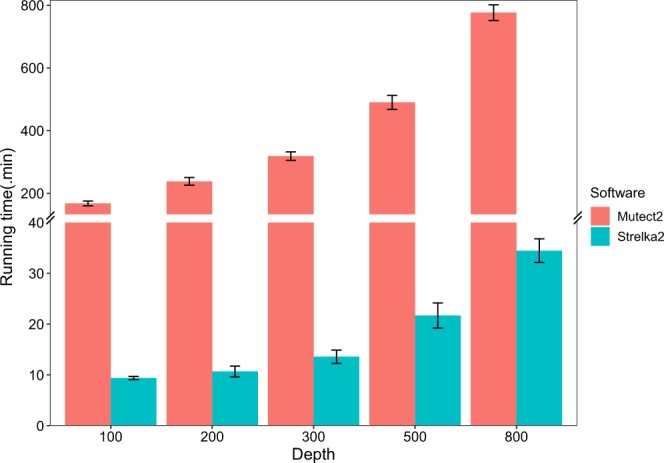
Software running time. The running time of Strelka2 and Mutect2 for each sample.

An important aspect of somatic variant calling is the time efficiency, especially when somatic variant calling is applied to clinical diagnosis. Therefore, we estimated the program running time in the previous somatic variant calling step. 60 GB memory size and 24 threads were allocated for both Mutect2 and Strelka2, finally, 180 running time data was collected. The detailed running time for each sample is presented in Supplementary Table 4. Figure 4 presented the runtime of Mutect2 and Strelka2, the average runtime of the samples with the same sequencing depth was calculated. Strelka2 took less than 10 minutes and less than 40 minutes to deal with 100X WES samples and 800X WES samples, respectively. Mutect2 took ~167 minutes and ~776 minutes to process 100X WES and 800X WES samples, respectively. In general, our results showed that Strelka2 was 17.8~22.6 times faster than Mutect2. It should be noted that 60 GB memory might not be enough for memory costing algorithms such as Mutect2, thus might influence the timings, a further timing study on the Mutect2 with the recommended environment would be interesting.

## Discussion

Many tools and pipelines have been developed to call somatic mutations, and several studies compared software and pipelines to assess the performance^21–24^. However, the accuracy of somatic mutation calling can be largely affected by sequencing depth and mutation frequency, which bring these researches limitations. Therefore, we performed a systematic comparison of performance on simulated tumor data of different sequencing depths and mutation frequency, as well as different somatic mutation calling tools. In our study, two standard DNA samples were used to conduct high-depth sequencing, and several pre-treatments were performed for down-sampling, then the files were down-sampled and mixed to simulate different sequencing and mutation frequency. After that, 30 combinations of sequencing depth and mutation frequency with 3 replicates each combination were generated, these files were then used to call somatic mutation by Strelka2 and Mutect2, respectively. Finally, 180 VCF files were produced for further assessment and comparison.

The tool used in data analysis is an important factor that influences the mutation calling performance. Our study showed that Mutect2 and Strelka2 performed similarly and both of these tools are useable, it was in concordance with a previous study that reported that EBCall, Virmid, Strelka and Mutect are the most reliable mutation callers for SNV calling, all of them performed well and similarly^21^. In addition, our study revealed that the advantage of Strelka2 was the higher recall and precision than Mutect2 when analyzing higher mutation frequency (≥20%) data, and its weakness was the lower recall rate and F-score than Mutect2 when mutation frequency was lower than 10%. It should be addressed that Strelka2 requires both normal and tumor sample for somatic mutation calling pipeline while Mutect2 can run in “tumor-only” mode, in the present study, Mutect2 was run in “normal-tumor” paired mode and the germline mutations from normal sample were excluded in the result, if Mutect2 run in “tumor-only” mode, only known germline mutation sites in databases will be excluded. In clinical studies, such “normal-tumor” paired strategy could be costly, thus it is interesting to find out if the “tumor-only” strategy could be a choice. Several studies have reported the performance of Mutect2 “tumor-only” mode and reported that tumor-only mutation detection resulted in a large number of false positive mutation sites^23,25^, thus may lead to inappropriate guidance for cancer therapies which is highly related to the patient safety and health care costs. Given these results, we did not perform the test between “tumor-only” mode and “normal-tumor” paired mode of Mutect2.

Our results showed that the mutation frequency also influenced the mutation calling performance. Across all sequencing depth and software, the performance of somatic mutation calling was better with 20~40% mutation frequency and worse with 1~10% mutation frequency. When mutation frequency was 1%, the recall rate was less than 35% across all sequencing depth and software, indicating that low-frequency mutation could not be well identified by the normal WES approach, improving on sequencing depth or other methods is needed to be applied. Furthermore, our results presented the influences of different sequencing depth for somatic mutation calling. Compared with 100X sequencing depth, increasing sequencing depth to 200X~800X could improve the recall rate and F-score by 0.6~44% and 0.02~0.45, respectively. Similar results have been reported by several studies that performed a comparison between two different sequencing depth and concluded that higher sequencing depth could improve somatic calling performance^19,21^. However, although 800X sequencing depth showed the best performance in our study, it is not recommended by us for the reason of high cost. Moreover, the drawback of an extreme high sequencing depth may also bring us new challenges. For example, the error rate of DNA polymerase and Illumina Hiseq sequencing platform are 10^−7^~10^−5^ and 0.2%, respectively. Besides, the PCR reaction of extreme high depth sequencing may exaggerate these errors and bring more false-positive results^26^. Hence, other methods providing high performance for detecting low frequency mutations are needed to be applied. Many error correction methods on WES have been developed and commonly used nowadays^27–33^, the unique molecular identifiers (UMIs) based error correction strategy is very useful in the above context. UMIs can tag sequences to help track molecules and remove errors in amplification and sequencing^34–38^, Michael *et al*. developed a method called “duplex sequencing” which tagged the sequencing adapter by a degenerated 12 randomized base (Duplex Tag) and 4 base length fixed sequence^27^. This approach can effectively classify the real mutation sites and PCR error thus can be useful for low-frequency mutation detection.

In addition to WES, amplicon sequencing and gene panel sequencing are two widely used methods in detecting mutations^39–41^. The advantages of gene panel and amplicon are that they require a lower initial sample concentration than WES and can easily achieve much higher sequencing depth, which enables them to detect gene relatively low-frequency somatic mutation, besides, gene panel and amplicon sequencing are cost-effective, makes them competitive in clinical diagnostic service^42–44^. However, gene panel and amplicon only target a small number of specific genes or short regions while WES can detect nearly all human exon regions^42–45^. Therefore, WES is used in the present study to detect enough mutation sites, which can help in obtaining a relatively accurate recall and precision value. In addition, the data analysis pipeline varies between WES, gene panel and amplicon sequencing. For WES data, a deduplication step is usually applied to remove PCR duplication, however, in gene panel and amplicon sequencing, the depth could be higher than 30000X and each molecule could have 10 PCR duplicates, the duplicates can be combined computationally into a consensus read, which helps sort out errors, thus the deduplication step is not needed^46^. Therefore, differences between pipelines may also influence the performance of mutation detection, thus it is interesting for future studies to focus on the performance and the comparison of different pipelines.

The shortcoming of this research is that we used a mixture of bam files instead of using the mixture of real-world DNA samples and send them for sequencing. However, since the sequencing or capture error of the experimental steps may be random, the reads quality control and filter should reduce experimental effects on mutation calling performance. As expected, different mutation frequency and sequencing depths had greater impacts on accuracy. Another point is the coverage bias introduced by mixing BAM files, thus we checked the coverage bias for 1~10% mutation frequency, the results showed that coverage bias was observed in sample with 100X and 1% mutation frequency, but in general, our mixed data is acceptable in coverage bias (see Supplementary Figs. S3 to S5).

In general, our study systematically evaluated the influence of different sequencing depths, mutation frequency and software, which will provide a comprehensive guidance for clinical somatic mutation research: using 200X sequencing depth for a relatively high mutation frequency (≥20%), applying other methods instead of using extreme sequencing depth for a relatively low mutation frequency (≤10%). In addition, since gene panel sequencing and amplicon sequencing have been successfully applied in clinical research, evaluating the two pipelines based on sequencing depth and mutation frequency systematically will be valuable for clinical practice in the future.

## Materials and Methods

### Sample preparation

Fifty μg each of NA12878 and YanHuang No.1 (YH-1) cell line genomic DNA were prepared after detecting their concentration by Qubit fluorometer 3.0 (Invitrogen), followed by the detection of purification using 1% Agarose Gel Electrophoresis. After sample purification, the genomic DNA was constructed as Illumina exome library. For library construction, 1 μg each of NA12878 and YH-1 genomic DNA was fragmented by Covaris E220 to DNA fragments with length of 100 to 500 bp, and then the Illumina adapter was ligated to both ends of each DNA fragment using SureSelectXT Reagent Kit (Cat No. G9611A, Agilent), followed by PCR amplification of each sample. In addition, the exome library was captured using the Human All Exon V5 Target Enrichment Baits (Cat No. 519-6216, Agilent).

### Sequencing data acquisition and data pretreatment

After library construction, all samples were sent to perform PE150 whole-exome sequencing (WES) on Illumina NovaSeq sequencing platform, with average sequencing depth was approximately 800X and 400X for NA12878 and YH cell line, respectively. Raw reads were first filtered and trimmed adapters using fastp (v0.19.4)^47^. After quality control, reads were mapped to the hg19 reference genome by BWA-MEM (v0.7.17)^48^ with the default parameter. Aligned SAM files were converted to BAM files and sorted by coordinate with Samtools (v1.7)^49,50^. The MarkDuplicate function of Picard (http://broadinstitute.github.io/picard, v2.18.11) was applied to remove duplicated reads of each bam file.

### Acquisition of true mutations set

All bam files from the previous steps were used to call true mutations set using Strelka2 (v2.9.7)^17^ and Genome Analysis Tool Kit HaplotypeCaller (v4.1.0.0)^51^. The germline mutation-calling pipeline with default parameters plus an “–exome” option of Strelka2 was conducted on both NA12878 and YH-1 data. After mutation calling, single nucleotide variation (SNV) sites were extracted and only sites with a “PASS” filter flag were kept. For GATK, mutation calling pipeline followed GATK’s best practice (https://software.broadinstitute.org/gatk/best-practices), and a hard filter (QD <2.0, FS >60.0, SOR >3.0, MQ <40.0, MQRankSum <−12.5, ReadPosRankSum <−8.0) was applied to both NA12878 and YH-1. For both Strelka2 and GATK, the SNVs which were homozygous in YH-1 but not in the absence of NA12878 were kept as true mutations set. The intersection of true mutation set which respectively derived from Strelka2 and GATK was considered as final true mutations set and used in the following analysis.

### Down-sampling, mixing bam files and calling mutations

Bam files which marked duplicates and sorted previously were down-sampled to different depths with Function DownsampleSam in Picard tools. The down-sampled bam files of NA12878 and YH-1 were merged by samtools to simulate tumor sample with different mutation frequency.

Both Strelka2 and GATK’s Mutect2 were used to call mutation according to their somatic pipeline, respectively. As recommended by Strelka2, the Manta variation caller (v1.5.0)^52^ was run first using the same parameters as Strelka2. And then the indel file derived from Manta was input into Strelka2 to help improve the precision of mutation calling, setting the parameter as default plus the “–exome” option. All SNVs with “PASS” filter flag were kept for the following evaluation. Besides, Mutect2 ran with default parameters except the “–disable-read-filter” option was set to “MateOnSameContigOrNoMappedMateReadFilter”, then a hard filter (QD <2.0, FS >60.0, SOR >3.0, MQ <40.0, MQRankSum <−12.5, ReadPosRankSum <−8.0) was applied to the SNV files.

### Evaluating mutation results

These SNVs were then compared with true mutations set using som.py, which is a somatic mutation evaluating tool in hap.py^53^ (https://github.com/Illumina/hap.py). The metrics we used to assess the performance were true positive (TP), false positive (FP), false negative (FN), precision, recall and F-score. The FP region was restricted to ± 10 bp from the true mutation sites. Precision rate, recall rate and F-score were defined as TP/(TP + FP), TP/(TP + FN) and 2*recall*precision/(recall + precision), respectively.

## Supplementary information

Supplementary Info.

Supplementary Table 1.

Supplementary Table 2.

Supplementary Table 3.

Supplementary Table 4.

## Data Availability

The raw sequencing reads and two deduplicated BAM files used to generate downsampled files are available in the NCBI sequence read archive (SRA) database (https://www.ncbi.nlm.nih.gov/bioproject/PRJNA549767). All the codes and scripts used to generate our data are available in GitHub (https://github.com/zic12345/SR2019).
