# Supplementary material for: Systematic comparison of somatic variant calling performance among different sequencing depth and mutation frequency
Source: Sci Rep. 2020 Feb 26;10:3501. doi: 10.1038/s41598-020-60559-5 (PMC7044309; doi:10.1038/s41598-020-60559-5)
Supplement: Supplementary file 1 — Supplementary Info. [file 41598_2020_60559_MOESM1_ESM.pdf]

Systematic comparison of somatic variant calling performance among different sequencing depth and mutation frequency

Zixi Chen[1], Yuchen Yuan[1], Xiaoshi Chen[1], Jiayun Chen[1], Shudai Lin[1], Xingsong Li[1], Hongli Du[1*]

[1] School of Biology and Biological Engineering, South China University of Technology, Guangzhou 510006, China

[*]Correspondence: hldu@scut.edu.cn; Tel.: +86-020-3938-0667

Supplementary Fig. S1: P-R curves of Strelka2 and Mutect2 in replicate group 2

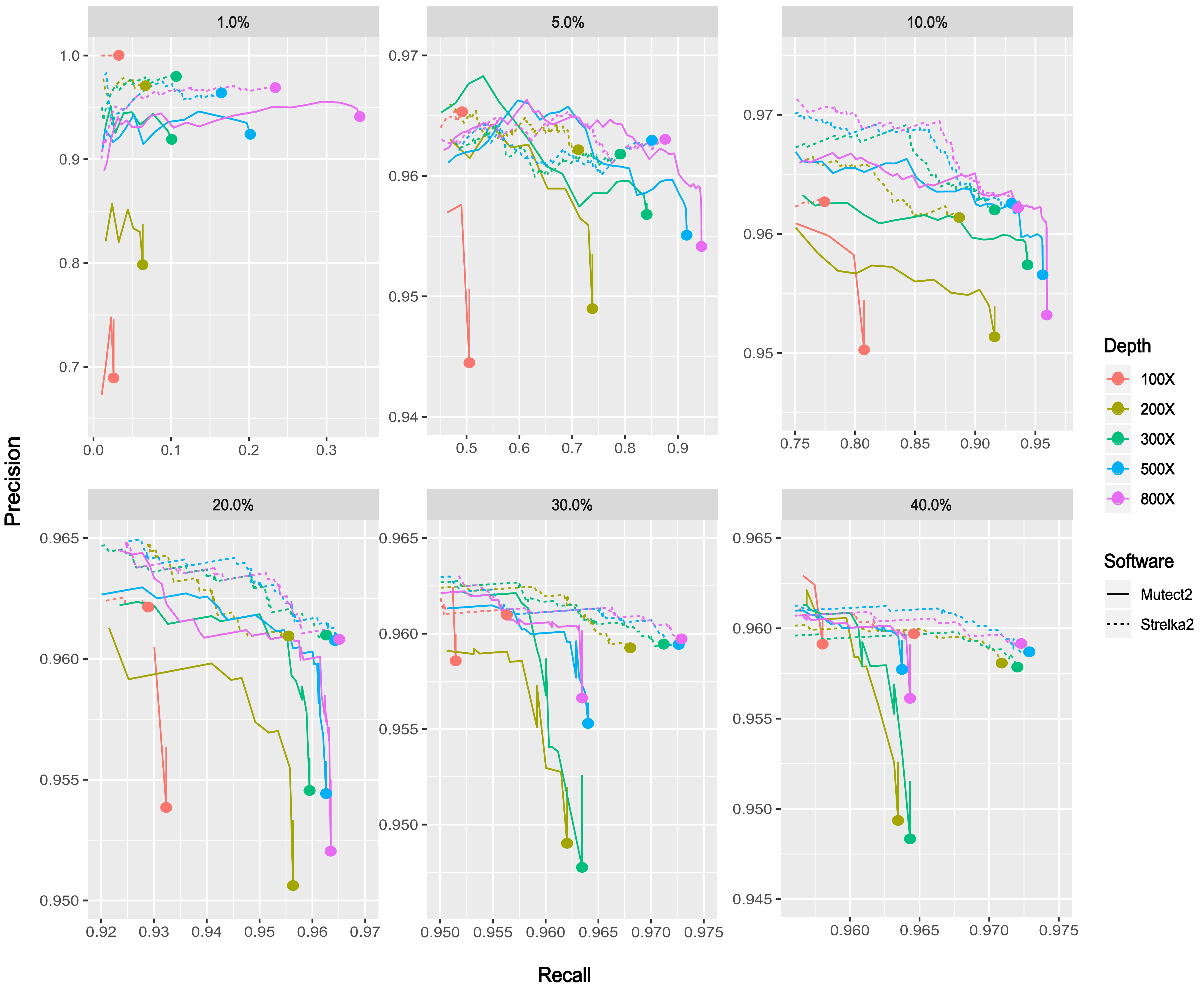

The P-R curves of replicate group 2. The colors in the figure represent different sequencing depths, the dotted lines represent Strelka2 and the solid lines represent Mutect2.

Supplementary Fig. S2: P-R curves of Strelka2 and Mutect2 in replicate group 3

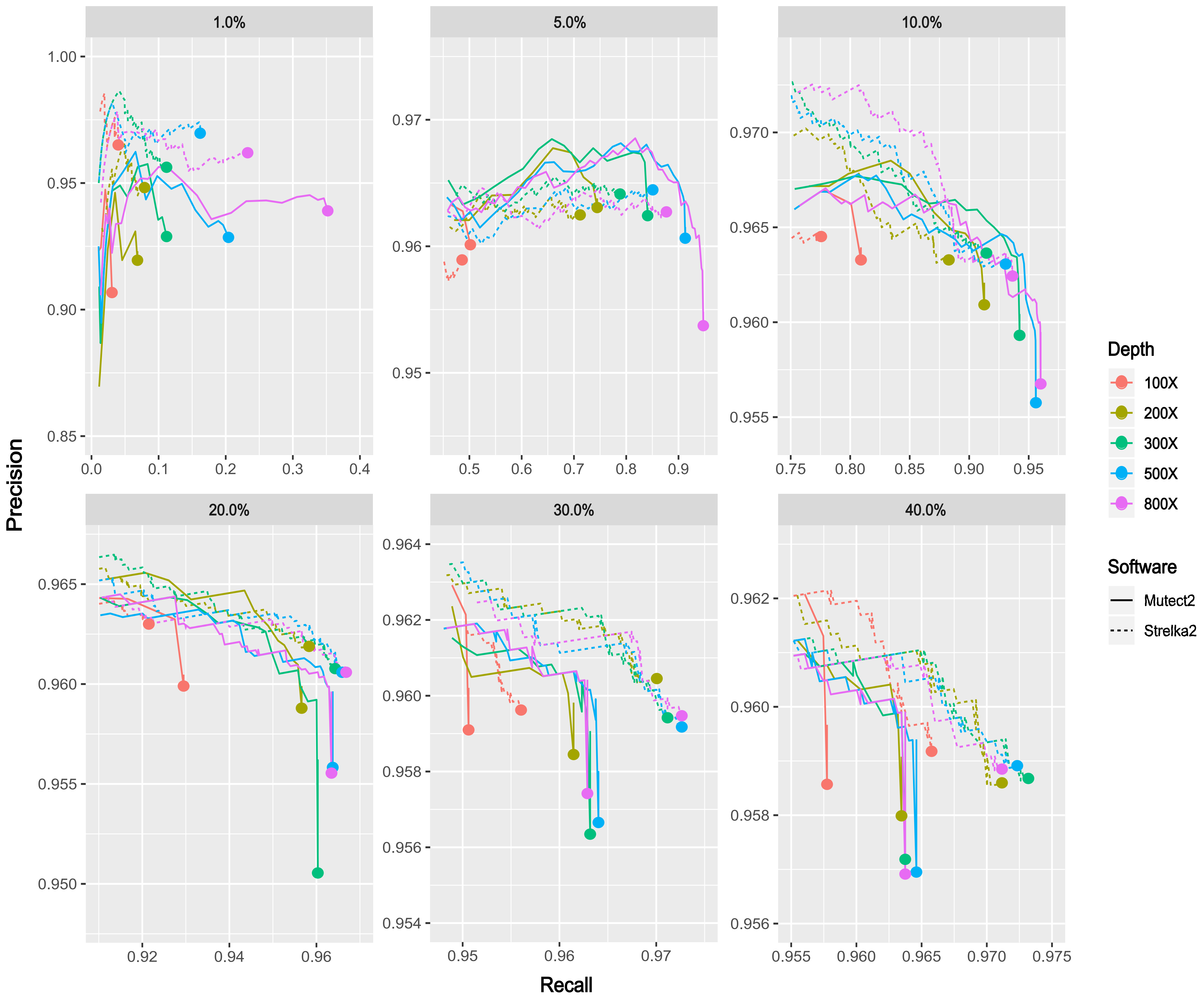

The P-R curves of replicate group 3. The colors in the figure represent different sequencing depths, the dotted lines represent Strelka2 and the solid lines represent Mutect2.

Supplementary Fig. S3: depth distribution of mutation sites in replicate group 1

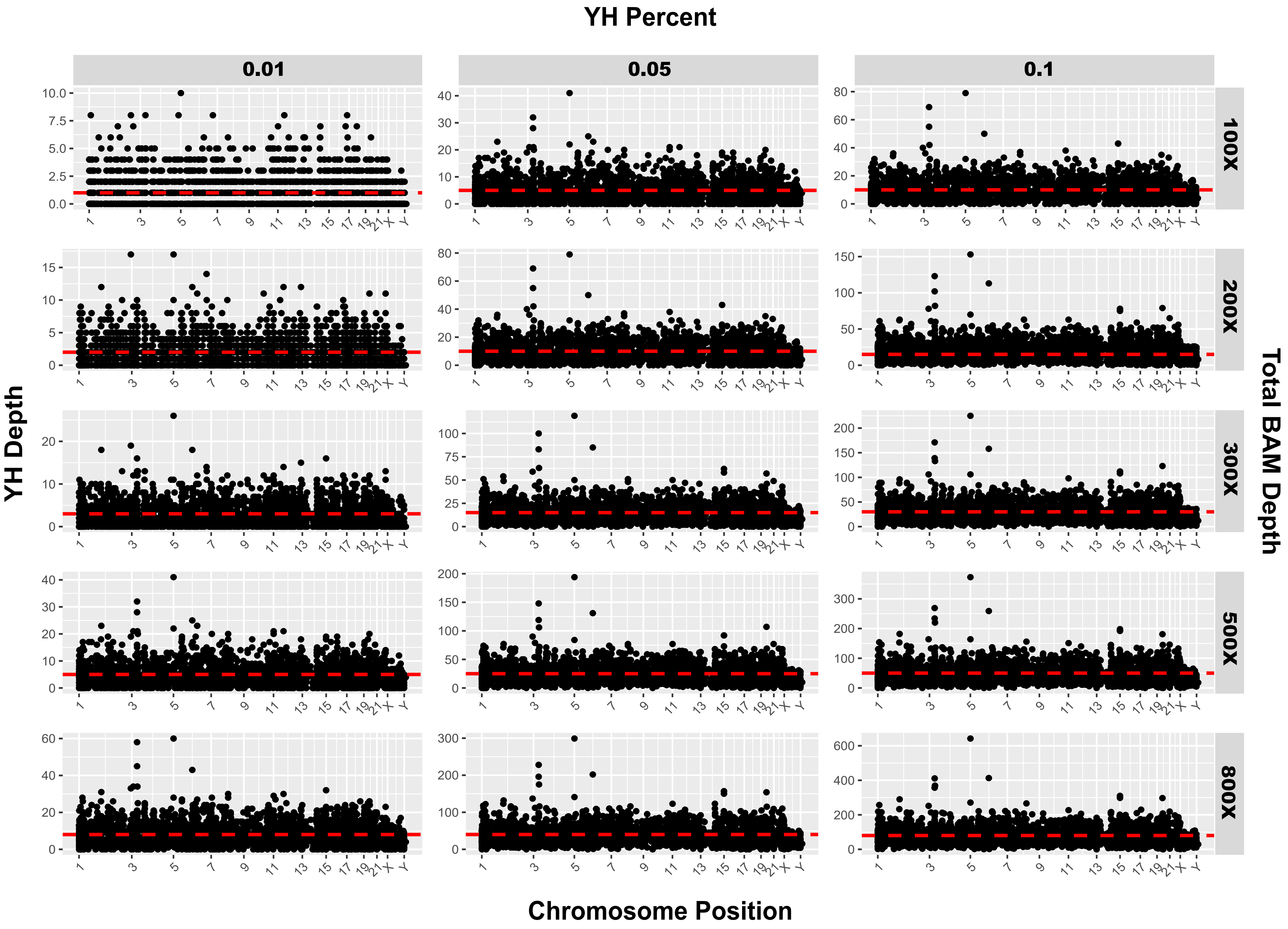

The depth distribution of the YH depth of mutation sites in replication group 1, the horizontal coordinates represent the mutation sites location in chromosome, vertical coordinates represents the YH depth of the mutation sites.

Supplementary Fig. S4: depth distribution of mutation sites in replicate group 2

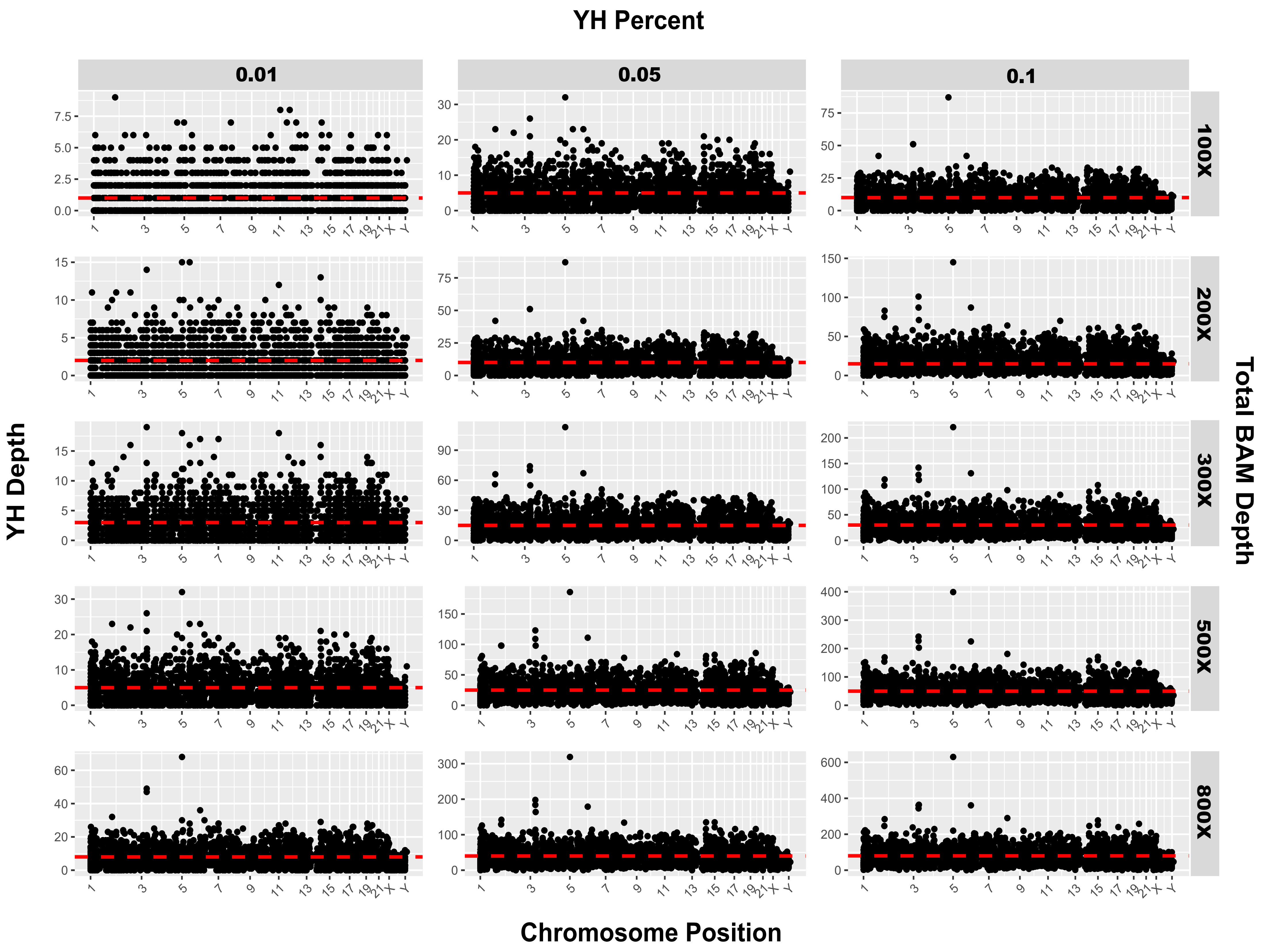

The depth distribution of the YH depth of mutation sites in replication group 2, the horizontal coordinates represent the mutation sites location in chromosome, vertical coordinates represents the YH depth of the mutation sites.

Supplementary Fig. S5: depth distribution of mutation sites in replicate group 3

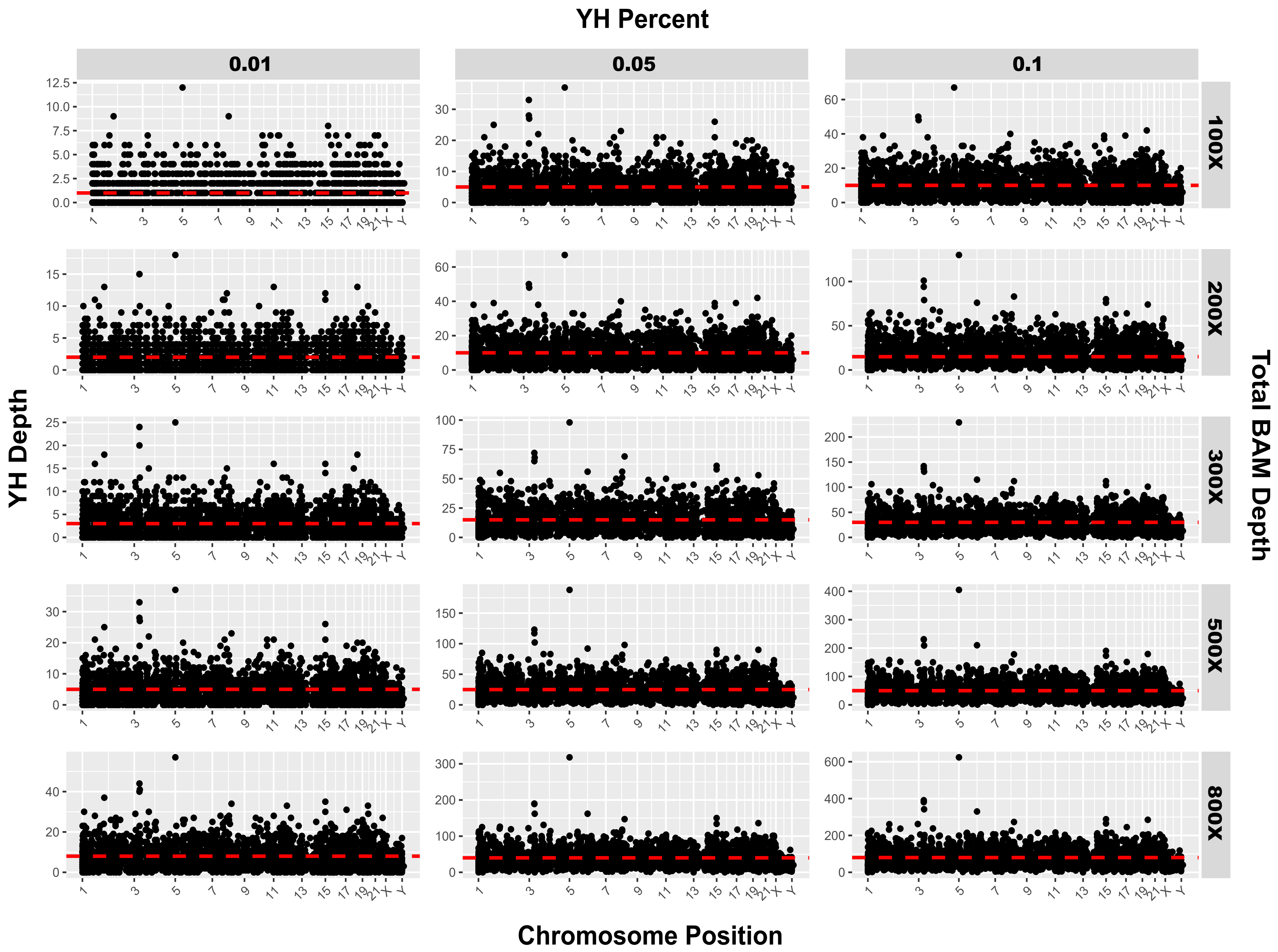

The depth distribution of the YH depth of mutation sites in replication group 3, the horizontal coordinates represent the mutation sites location in chromosome, vertical coordinates represents the YH depth of the mutation sites.
